# Effects of One-Year Menaquinone-7 Supplementation on Vascular Stiffness and Blood Pressure in Post-Menopausal Women

**DOI:** 10.3390/nu17050815

**Published:** 2025-02-27

**Authors:** Femke de Vries, Rudolf Bittner, Katarzyna Maresz, François Machuron, Olav Gåserød, Jean-François Jeanne, Leon J. Schurgers

**Affiliations:** 1 Department of Biochemistry, Cardiovascular Research Institute Maastricht (CARIM), Maastricht University, P.O. Box 616, 6200 MD Maastricht, The Netherlands; 2 Research and Development Department, Gnosis by Lesaffre, Lesaffre International, 59700 Marcq-en-Baroeul, France; 3 Independent Researcher, 30-376 Krakow, Poland; 4 Data Science & Computational Biology Department, Lesaffre International, 59700 Marcq-en-Baroeul, France; 5 Research and Application Department, Gnosis by Lesaffre, Lesaffre International, 59700 Marcq-en-Baroeul, France; 6 Institute of Experimental Medicine and Systems Biology, RWTH Aachen University, 52074 Aachen, Germany

**Keywords:** pre/peri-menopause, post-menopause, vitamin k homeostasis, vascular stiffness, blood pressure

## Abstract

**Background/Objectives**: Post-menopausal women are at an increased risk of developing cardiovascular disease. Menaquinone-7 (MK-7) is a fat-soluble vitamin involved in coagulation and maintaining vascular health. The aim of the post hoc analysis of this one-year study is to investigate the effects of MK-7 supplementation on the vascular parameters in pre-, peri-, and post-menopausal women. **Methods**: In a clinical intervention trial (NCT02404519), a total of 165 women with a low vitamin K status received either 180 µg of MK-7 daily (*n* = 82) or a matching placebo (*n* = 83) for one year. Established vascular parameters were measured before and after one year of vitamin K2 supplementation. Pre-, peri-, and post-menopausal women were subdivided according to arterial stiffness, with a high b-stiffness index defined as being greater than the overall median of 9.83. **Results**: The post hoc analyses showed a significant decrease in desphospho-uncarboxylated matrix Gla protein (dp-ucMGP) plasma levels after MK-7 supplementation (pre/peri, *p* = 0.009; post, *p* < 0.001). MK-7 treatment significantly attenuated vascular stiffness in post-menopausal women (placebo +49.1% ± 77.4; MK-7 +9.4% ± 67.1; *p* = 0.035). Post-menopausal women with a high stiffness index showed significantly improved vascular markers after MK-7 treatment, e.g., a decreased blood pressure at brachialis (−3.0% ± 9.0; *p* = 0.007) and an increased distensibility coefficient (+13.3% ± 32.3; *p* = 0.040). **Conclusions**: Our results confirm that menopause affects vascular health status. Post-menopausal women with an increased stiffness benefit most from MK-7 supplementation, with a significantly improved blood pressure. Further research is needed to unravel the beneficial effects of MK-7 in post-menopausal women.

## 1. Introduction

Over the years, the role of vitamin K hemostasis has expanded from coagulation to effects on arterial calcification, bone formation, and the regulation of cell growth [1]. Vitamin K is a fat-soluble vitamin that serves as a cofactor for the carboxylation of vitamin-K-dependent proteins. One such protein is matrix Gla protein (MGP), which is primarily synthesized by vascular smooth muscle cells and chondrocytes. When secreted into the extracellular space, MGP acts as a local inhibitor of vascular calcification. A poor vitamin K status, which can be measured by plasma desphospho-uncarboxylated MGP (dp-ucMGP), is associated with an increased risk of several types of diseases, including cardiovascular disease (CVD), hypertension, and osteoporosis [1]. Data have shown that dp-ucMGP levels rise during aging [2], reflecting a vitamin K deficiency [3]. In line with these data, an increase in dp-ucMGP levels is associated with a higher risk for vascular calcification, vascular stiffness, and osteoporosis [3]. During menopause, bone density has been shown to decrease significantly. Estrogen plays a key role in regulating osteoblasts and osteoclasts, the cells necessary for maintaining healthy bone tissue. As estrogen levels decrease during menopause, bone resorption increases and bone formation decreases, resulting in a loss of bone density over time. Vitamin K supplementation, specifically menaquinone-7 (MK-7; K2), has been shown to improve bone density and cardiovascular health by activating vitamin-K-dependent proteins (VKDP), including osteocalcin and MGP [4,5,6]. However, the degree of carboxylation of VKDPs by vitamin K supplementation varies among different populations [5,7].

Vascular stiffness significantly contributes to the development of CVD. Its origin is multifaceted, and they are intertwined with each other, including a reduced elastin/collagen ratio, reactive-oxygen-species-induced inflammation, calcification, and vascular smooth muscle cell phenotype switching [8]. Hereby, reactive oxygen species can not only induce inflammation, but also be a result of inflammation, enhancing the inflammation phenotype of vascular smooth muscles. Although CVD affects men and women equally, women often present clinical phenotypes later in life [9]. Sex hormones, such as estrogen and progesterone, play cardiovascular protective roles in women. Their levels decrease during menopause, resulting in an increase in dp-ucMGP levels [6,7]. Consequently, post-menopausal women are more prone to hypertension and vascular stiffness, accelerated vascular aging, and risk for CVD.

The aim of the post hoc analysis of this one-year follow-up study is to elucidate the effect of MK-7 supplementation on the vascular parameters in pre-, peri-, and post-menopausal women. The study builds on a previous three-year clinical trial that provided initial insights into the potential benefits of MK-7 for vascular health. The one-year study specifically enrolled individuals with a low extrahepatic vitamin K status, indicating a higher cardiovascular risk. This follow-up analysis aims to confirm and further investigate the long-term impacts of vitamin K2 supplementation on the vascular stiffness and overall cardiovascular health in these high-risk populations.

The previous three-year clinical trial demonstrated the potential benefits of MK-7 for vascular health, showing statistically significant results after three years in post-menopausal women [4]. Building on these findings, the subsequent one-year study specifically enrolls women and men with a low extrahepatic vitamin K status, a group indicating a higher cardiovascular risk. By targeting individuals with a poor vascular vitamin K status, the study aims to achieve quicker results, potentially providing a protocol for MK-7 intervention studies that can deliver cardiovascular outcomes within one year. This follow-up confirms the impact of MK-7 supplementation on the vascular stiffness and overall cardiovascular health in high-risk populations [10].

The aim of the post-hoc analysis of this one-year follow-up study is to elucidate the effects of MK-7 supplementation on the vascular parameters in pre-, peri-, and post-menopausal women with a low extrahepatic vitamin K status, indicating a higher cardiovascular risk.

## 2. Materials and Methods

### 2.1. Study Population

This double-blind, placebo-controlled clinical intervention trial, including 243 volunteers, both men and women, was performed by Maastricht University between 2015 and 2018. Written informed consent was obtained from all subjects before entering the study. Details on recruitment and the participants have been described previously [10]. The required sample size was determined based on MK-7-induced changes in the carotid-femoral pulse wave velocity (cfPWV) in a previous study [4]. Included were subjects between the age of 40 and 70 years, with a BMI between 20 and 35 kg/m^2^, of Caucasian race, and with circulating dp-ucMGP > 400 pmol/L, which represented a low extrahepatic vitamin K status and increased cardiovascular risk. Excluded were subjects suffering from cardiovascular disease, a blood coagulation disorder, hyperlipidaemia, a history of metabolic or gastrointestinal disease, the consumption of more than 3 units of alcoholic beverages per day, and the use of estrogen replacement, corticosteroids, anticoagulants, or vitamin-K-containing dietary supplements. This study was conducted according to the guidelines laid down in the Declaration of Helsinki, and all procedures involving human subjects were approved by the Medical Ethics Committee of the Maastricht University (Maastricht, The Netherlands, code: METC143058; date: 1 January 2016).

### 2.2. Study Design

After randomization, the participants received capsules with 180 mcg MK-7 (MenaQ7, Nattopharma AS, Norway—now part of Gnosis by Lesaffre, France) (*n* = 121) or a matching placebo (*n* = 122) to be consumed once a day during breakfast or dinner. During the entire study period of 12 months, the subjects were asked to stick to their normal diet. Blood was taken at intake (inclusion/exclusion), at baseline, and at the end of the study by venipuncture to prepare ethylenediaminetetraacetic acid (EDTA) plasma. Of the 166 women that participated in the clinical study (NCT02404519), a total of 165 women were selected and divided into pre/peri-menopausal women (*n* = 78) and post-menopausal women (*n* = 87). One participant was excluded due to a missing post-menopausal status. Menopause was confirmed in post-menopausal women 12 months after their last menstrual period.

### 2.3. Biomarkers

Dp-ucMGP plasma levels were measured using an Enzyme-Linked Immune Sorbent Assay (ELISA) test, as described before [11]. The intra- and inter-assay variations were 7.6 and 6.8%, respectively, and the lower detection limit was 50 pmol/L. Paired samples (baseline and endpoint plasma samples) of each subject were assessed on the same ELISA plate to minimize inter-assay variation.

### 2.4. Regional Arterial Stiffness

Regional carotid-femoral (cf-PWV) and carotid-radial (cr-PWV) pulse wave velocities were assessed non-invasively by using mechanotransducers directly applied on the skin (Complior, Artech Medical, Pantin, France) [12]. The pulse wave velocity (PWV) was used to assess arterial stiffness at both the central and peripheral arteries [12]. cf-PWV was measured to assess central arterial stiffness and cr-PWV was measured to assess peripheral arterial stiffness. The measurement of PWV occurred at baseline and after 1 year of supplementation. Internal yearly Quality Control assessments for the PWV measurements showed coefficients of variation of less than 10%.

### 2.5. Carotid Artery Ultrasound Examination and Arterial Blood Pressure Measurement

The vessel wall characteristics of the common carotid artery were measured with ultrasound. Echotracking was performed as described previously. In short, a 7.5 MHz linear array transducer was connected to an ultrasound scanner (MyLab One, Esaote, Maastricht, The Netherlands). The following variables were measured or calculated: intima–media thickness, arterial diameter and distensibility (change in diameter from diastole to systole), arterial distensibility coefficient and compliance coefficient, Young’s elasticity modulus E, stiffness index β, and local PWV carotid artery. Yearly Quality Control (inter- and intra-operator CVs) assessments for the measurements of the local arterial stiffness showed coefficients of variation of less than 10%.

Arterial blood pressure was recorded at the level of the brachial artery by the means of a semi-automated oscillometric device (DINAMAP). Blood pressure was measured at baseline and after 1 year of treatment.

### 2.6. Statistical Analyses

Baseline parameters were compared between the two groups of menopausal women (pre/peri- versus post-menopausal) with T-tests in the case of data normality. When this assumption was rejected, Wilcoxon signed tests were used. The evolution of cardiovascular parameters between baseline and the final timepoint (one year of supplementation with placebo or MK-7) was calculated using relative difference from baseline (expressed in %). The effect of MK-7 supplementation versus the placebo on relative differences was appreciated within the two menopausal groups using Analysis of Variance (ANOVA) models with menopausal status and treatment as the main effects and the interaction term to study if the treatment effects were heterogenous within the two menopausal groups. ANOVA assumptions were checked (normality of residuals and equal variances). In the case of a violation of one of these assumptions, data were log-transformed using the formula with min: minimum in order to consider negative values. Treatment effects on the evolution of cardiovascular parameters within the menopausal groups were appreciated using adjusted *p*-values (using the Benjamini–Hochberg method to control the false discovery rate). The same analysis was performed for the study of the stiffness index (SI) group effects on the cardiovascular parameters within the two treatment groups. In this aim, all studied patients were separated into two groups based on the position of their stiffness index at baseline compared with the overall SI median (9.83). Analyses were performed with R statistical programming language version 4.4.2 (R Foundation for Statistical Computing, Vienna, Austria). The various characteristics and their abbreviations are described with corresponding equations (if applicable) in Abbreviations Section. 

## 3. Results

This double-blind, placebo-controlled clinical intervention trial (NCT02404519) included a total of 243 apparently healthy men and women between 40 and 70 years of age. For the post hoc analysis, 166 women with baseline dp-ucMGP levels greater than 400 pmol/L were selected. One woman was excluded due to missing menopausal status information. The baseline characteristics of the remaining participants are summarized in Table 1.

The post hoc analysis cohort included 78 pre/peri-menopausal women (47.3%) and 87 post-menopausal women (52.7%). The pre/peri-menopausal and post-menopausal women showed no significant difference in their BMI, weight, or height. As expected, the post-menopausal women were older compared to the pre/peri-menopausal women. At baseline, the vascular parameters were significantly increased in the post-menopausal women compared to the pre/peri-menopausal women, including the intima–media thickness (*p* = 0.013), carotid artery diameter (*p* = 0.007), caPWV (*p* = 0.012), and blood pressure at the carotid artery (*p* = 0.018).

An intervention with MK-7 was conducted, whereby pre/peri/post-menopausal women received 180 mcg of MK-7 daily (*n* = 82) or a matching placebo (*n* = 83) for one year. Post hoc analysis showed a significant decrease in the dp-ucMGP plasma levels of MK-7-supplemented pre/peri-menopausal (*n* = 34; −31.5% ± 16.6; *p* < 0.001) and post-menopausal women (*n* = 48; −27.5% ± 22.1; *p* < 0.001) (Table 2). Furthermore, MK-7 supplementation showed a trend regarding distensibility (pre/peri −4.9 ± 21.8; post +2.8 ± 30.8; *p* = 0.037) and compliance (pre/peri −7.4 ± 21.4; post +2.2 ± 30.8, *p* = 0.020). Moreover, placebo-supplemented women showed an opposite trend, whereby distensibility and compliance decreased further over the supplementation period.

**Figure 1:**
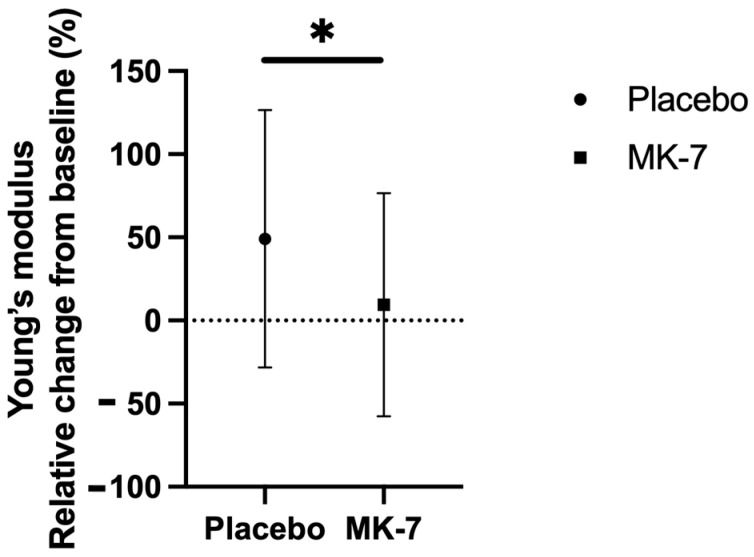
Relative change from baseline in Young’s modulus within treatment groups in post-menopausal women population (mean ± standard error). Adjusted *p*-value is depicted using Benjamini–Hochberg method. * *p*-value < 0.05.

MK-7 treatment significantly attenuated vascular stiffness in post-menopausal women (Young’s modulus; placebo +49.1% ± 77.4; MK-7 +9.4% ± 67.1; adjusted *p*-value = 0.035, Figure 1). Due to significant alterations in vascular stiffness, both groups (pre/peri- and post-menopausal women) were further divided according to a low or high stiffness index (high b-stiffness index: >overall median 9.83). Blood pressure, both brachialis (±6.0% ± 9.4; *p* = 0.005) and carotid artery (±6.0% ± 9.4; *p* = 0.004), was significantly increased after the placebo intervention in pre/peri-menopausal women with a high stiffness index (Table 3).

Pre/peri-menopausal women with a high vascular stiffness that received placebo supplementation showed a significantly increased blood pressure, both at brachialis (low stiffness, −2.8% ± 4.7; high stiffness, +6.0% ± 9.4; *p* = 0.005) and carotid artery (low stiffness, −2.8% ± 4.6; high stiffness, +6.0% ± 9.4; *p* = 0.004). There was no significant difference observed in vascular markers (diameter of the carotid artery (*p* = 0.084); blood pressure at brachialis (*p* = 0.85); or blood pressure at carotid artery (*p* = 0.81) in post-menopausal women with a high stiffness index that received a placebo for one year.

Post-menopausal women with a high stiffness index at baseline showed significantly improved vascular markers after MK-7 treatment, e.g., decreased blood pressure at brachialis (low SI, +6.3% ± 10.4; high SI, −3.0% ± 9.0; *p* = 0.007), decreased blood pressure at carotid artery (−4.49% ± 11.66; *p* = 0.008), an increased distensibility coefficient (low SI, −9.7% ± 24.0; high SI, +13.3% ± 32.3; *p* = 0.013), and an increased compliance coefficient (low SI, −10.3% ± 23.6; high SI, ±12.7% ± 32.6; *p* = 0.012) (Table 4). No significant improvement could be observed for intima–media thickness, however, when compared to post-menopausal women with a low stiffness index, a high stiffness index attenuated intima–media thickness (low SI, −3.3% ± 11.4; high SI, −0.8% ± 6.9; *p* = 0.48). No significant results were obtained after MK-7 treatment in pre/peri-menopausal women.

## 4. Discussion

The purpose of this study was to investigate the effects of MK-7 supplementation on the vascular stiffness in pre/peri-menopausal versus post-menopausal women. Our findings confirm that menopause adversely affects vascular health, leading to an increased vascular stiffness in post-menopausal women. Notably, post-menopausal women with a heightened arterial stiffness showed the greatest benefit from MK-7 supplementation, experiencing significant improvements in their blood pressure and vascular compliance.

Expanding upon the findings of the three-year clinical study demonstrating the cardiovascular benefits of MK-7 supplementation in post-menopausal women [4], this subsequent one-year study focused specifically on enrolling individuals—both women and men—with a low extrahepatic vitamin K status, a demographic associated with an increased cardiovascular risk. This follow-up confirmed the impact of MK-7 supplementation on the vascular stiffness and overall cardiovascular health in high-risk populations [10].

Our post hoc analysis focused exclusively on women exhibiting a low extrahepatic vitamin K status, a group known for a heightened cardiovascular risk that is typically underrepresented in clinical settings. This allowed us to evaluate the impact of MK-7 supplementation on clinical outcomes over one year. A low vitamin K status leads to uncarboxylated, inactive MGP, thereby elevating the risk of vascular calcification and stiffness. The cut-off value of 400 pmol/L in our study was based on the FLEMENGHO study, which showed that dp-ucMGP predicts non-cancer and cardiovascular mortality. The reference stated that dp-ucMGP levels should be maintained below 437 pmol/L. Ikari et al. showed that the benefits of vitamin K2 supplementation (administered as MK-4) only benefited individuals with low vitamin K levels at baseline, highlighting the importance of initial vitamin K levels [13]. Our study aligns with those results, demonstrating that MK-7 supplementation is particularly beneficial for post-menopausal women with a low vitamin K status. Our findings confirm that menopause adversely affects vascular health, leading to an increased vascular stiffness in post-menopausal women. Notably, post-menopausal women with a heightened arterial stiffness showed the greatest benefit from MK-7 supplementation, experiencing significant improvements in their blood pressure and vascular compliance.

A recent systematic review showed that vitamin K2 supplementation significantly reduces uncarboxylated VKDPs, e.g., dp-ucMGP. Furthermore, a reduction in vascular calcification was obtained, with a trend toward improving vascular stiffness [14]. In line with this review, other studies have consistently linked reduced vitamin K intake and elevated levels of inactive MGP (dp-ucMGP levels) to an increased risk of cardiovascular mortality [3,15,16,17]. Our study is in line with these results and shows that MK-7 supplementation reduces dp-ucMGP in pre-, peri-, and post-menopausal women.

Age is an important risk factor for CVD, especially in women. Age is negatively correlated with aortic distensibility, an important vascular biomarker for cardiovascular risk [18]. Furthermore, aortic distensibility has been identified in individuals with metabolic dysfunction or peripheral vascular disease [18]. Our study provides insight into age-related cardiovascular risks, whereby post-menopausal women exhibit significant differences in aortic distensibility compared to pre/peri-menopausal women. Moreover, the distensibility coefficient showed an inverse correlation with the stiffness index in post-menopausal women. These findings support the well-established notion that menopausal status has a significant impact on various cardiovascular parameters [14,19]. Furthermore, our study provides support for previous MK-7 supplementation studies on vessel wall stiffness in post-menopausal women [4,10]. Knapen et al. showed that MK-7 results in reduced levels of circulating inactive MGP (dp-ucMGP levels), leading to improvements in the elastic properties of the carotid artery. Additionally, it has been shown that MK-7 intake (180 mcg/day) is able to decrease age-related vascular stiffness in individuals with a low vitamin K status at baseline during a one-year supplementation period [10]. Our study extends both study results by demonstrating that one year of MK-7 supplementation not only improves vascular stiffness, but also reduces blood pressure in post-menopausal women with a reduced vitamin K status and high vascular stiffness.

Although our study included a great sample size, these sample sizes decreased when subcategorized based on vascular stiffness and MK-7 dosage applied. When comparing 180 mcg MK-7 versus placebo, PWV did not significantly differ in post-menopausal women. However, there was a decrease observed in PWV, indicating that healthy post-menopausal women with a low initial vitamin K status could possibly benefit more from a higher dose of MK-7 supplementation. Different doses of vitamin K have been tested in healthy volunteers, resulting in an improvement in vitamin K status [6]. Therefore, future research will be essential to investigate the optimal dosage of MK-7 per individual to implement individual nutrition recommendations. Additionally, our study only tested the impact of vitamin K2 as a standalone. However, research has shown a positive effect of combination therapy with vitamin K and vitamin D. Several studies have shown the association of low vitamin K and D levels with cardiovascular health [20,21,22]. Braam et al. showed that only individuals supplemented with vitamin K1 and vitamin D3 saw a beneficial effect on the elastic properties of their arterial vessel wall [20]. In this study, a combination of vitamin D and MK-7 was employed alongside calcium, magnesium, and zinc, making it challenging to isolate the specific impacts of vitamin D and K. Furthermore, a 2-year study that included 365 elderly men suffering from aortic valve calcification (AVC) highlighted that a combination of MK-7 and vitamin D did not influence AVC progression [23]. This emphasizes gender specificity and preventive vitamin supplementation. Additionally, vitamin K and D are also important for bone health. Ueki et al. found a significant impact on vertebral bone mass in post-menopausal women when they were supplemented with MK-7 and vitamin D3. Overall, a synergistic effect of vitamin K2 and vitamin D is suggested in the literature. Therefore, additional research observing the combinational supplementation of vitamin K and vitamin D in post-menopausal women would give more insight regarding the benefits of combination therapy.

## 5. Conclusions

In conclusion, we confirm that a post-menopausal status has a significant impact on various vascular parameters. Furthermore, post-menopausal women have a significantly higher blood pressure, higher vascular stiffness, increased intima–media thickness, and enlarged diameter of the carotid artery compared to pre/peri-menopausal women. These results show the importance of cardiovascular research especially targeting women, an underrepresented group in cardiovascular clinical trials. Examining cardiovascular health before and after menopause is essential for developing effective prevention and treatment strategies that can significantly improve the cardiovascular outcomes and overall well-being of women during aging. Supplementation with MK-7 decreases these cardiovascular parameters, suggesting an improvement in cardiovascular health. Furthermore, post-menopausal women with a high vascular stiffness at baseline showed greater benefits from MK-7 supplementation regarding their blood pressure and vascular stiffness. This study supports previous findings and adds insight about the contributing risk factors of CVD, e.g., a low extrahepatic vitamin K status. Recognizing these risk factors and acknowledging the benefit of MK-7 supplementation are crucial for tailoring cardiovascular risk assessment, prevention, and management strategies to address the specific needs of women at different stages of menopause. Further, confirmatory studies with larger samples sizes are needed to define dietary recommendations for MK-7.

## Figures and Tables

**Table 1:** Baseline characteristics of patients by menopausal status.

Baseline Parameter ^1^	Pre/Peri-Menopausal Women *n* = 78	Post-Menopausal Women *n* = 87	*p*-Value
Age	**57.09 (52.91; 63.53)**	**64.10 (59.83; 67.45)**	**<0.001**
Height	1.65 (1.62; 1.70)	1.66 (1.61; 1.69)	0.85
Weight	67.55 (61.3; 77.03)	69.7 (61.75; 78.5)	0.69
BMI	25.51 ± 3.91	25.66 ± 3.79	0.91
Dp-ucMGP	624 (513; 741)	617 (519; 818)	0.75
Intima–media thickness	**649 (556; 699)**	**671 (604; 794)**	**0.013**
Diameter of the carotid artery	**6.82 ± 0.64**	**7.11 ± 0.73**	**0.007**
Blood pressure at brachialis systole	**119.7 ± 13.3**	**125.0 ± 13.1**	**0.011**
Blood pressure at brachialis diastole	70.3 ± 6.9	70.6 ± 8.3	0.80
Local pulse wave velocity carotid artery	6.28 (5.8; 7.36)	6.97 (6.26; 7.94)	**0.012**
Distensibility coefficient	**24.0 (17.5; 28.2)**	**19.9 (15.2; 24.3)**	**0.013**
Compliance coefficient	0.91 ± 0.33	0.87 ± 0.40	0.51
Stiffness index	**9.92 ± 3.55**	**11.02 ± 3.38**	**0.045**
Blood pressure at carotid artery systole	**108.8 ± 13**	**113.7 ± 13.6**	**0.018**
Blood pressure at carotid artery diastole	70.4 ± 6.9	70.6 ± 8.2	0.82
Young’s modulus	0.57 ± 0.27	0.56 ± 0.24	0.67
PWV ^2^ between carotid artery and radialis	11.18 ± 1.32	10.92 ± 1.33	0.23
PWV ^2^ between carotid artery and femoralis	10.85 ± 2.17	10.46 ± 2.09	0.25

^1^ Depending on normality, data are presented with mean ± SD or median (Q1; Q3). ^2^ Pusle wave velocity.

**Table 2:** Pre/peri- and post-menopausal women undergoing MK-7 treatment versus placebo.

Cardiovascular Parameter ^1^	Pre/Peri Menopausal Women			Post-Menopausal Women			Int *p* ^5^
	Placebo *n* = 44	MK-7 *n* = 34	*p* ^4^	Placebo *n* = 39	MK-7 *n* = 48	***p* ^4^**	
**dp-ucMGP**	**+8.0 ± 21.5**	**−31.5 ± 16.6**	**<0.001**	**+4.2 ± 23.1**	**−27.5 ± 22.1**	**<0.001**	0.26
Intima–media thickness	+1.7 ± 10.5	−2.1 ± 13.2	0.42	−0.5 ± 9.8	−1.6 ± 9.4	0.73	0.45
Diameter of the CA ^2^	+0.3 ± 4.3	−0.05 ± 3.4	0.69	+1.1 ± 4.7	−0.4 ± 3.4	0.51	0.38
Blood pressure at brachialis syst	−1.7 ± 17.2	+1.7 ± 8.6	0.47	+2.5 ± 8.8	+1.4 ± 10.6	0.91	0.24
Blood pressure at brachialis diast	+1.1 ± 8.3	+3.3 ± 9.6	0.89	+2.6 ± 9.0	+2.7 ± 8.6	0.96	0.47
Local pulse wave velocity CA ^2^	+3.9 ± 16.9	+4.5 ± 12.0	0.85	+8.6 ± 14.4	+1.9 ± 16.1	0.35	0.14
**Distensibility coefficient**	−0.2 ± 31.9	−4.9 ± 21.8	0.55	−11.4 ± 22.5	+2.8 ± 30.8	0.16	**0.037**
**Compliance coefficient**	−0.3 ± 30.5	−7.4 ± 21.4	0.40	−11.8 ± 24.6	+2.2 ± 30.8	0.17	**0.020**
**Young’s modulus**	+9.5 ± 57.8	+11.9 ± 67.8	1.00	**+49.1 ± 77.4**	**+9.4 ± 67.1**	**0.035**	0.056
Stiffness index	+6.4 ± 33.0	+1.3 ± 22.1	0.61	+7.9 ± 24.6	+1.0 ± 24.4	0.61	0.83
Blood pressure at CA ^2^ syst	+1.9 ± 11.5	+4.5 ± 13.5	0.61	+3.6 ± 8.9	+0.1 ± 11.8	0.48	0.091
Blood pressure at CA ^2^ diast	+1.1 ± 8.3	+3.2 ± 9.7	0.91	+2.6 ± 9.0	+2.8 ± 8.7	0.91	0.51
PWV ^3^ between CA ^2^ and radialis	−0.04 ± 13.9	−1.7 ± 10.5	0.67	+3.6 ± 11.0	+0.1 ± 11.3	0.40	0.62
PWV ^3^ between CA ^2^ and femoralis	+4.0 ± 15.5	+4.9 ± 25.4	0.85	+11.2 ± 19.6	+1.8 ± 16.5	0.20	0.11
Bone mineral density total body	+0.2 ± 1.7	−0.04 ± 1.7	0.89	−1.9 ± 9.8	−1.0 ± 7.9	0.66	0.61

^1^ Data are presented as relative change from baseline (in %). ^2^ Carotid artery. ^3^ Pulse wave velocity. ^4^ *p*-values for adjusted for multiplicity testing with Benjamini–Hochberg method. ^5^ Interaction *p*-value from ANOVA model with menopausal status and treatment as fixed effect.

**Table 3:** Cardiovascular parameters’ evolution in women supplemented with placebo.

Cardiovascular Parameter ^1^	Pre/Peri-Menopausal Women			Post-Menopausal Women			Int *p* ^6^
	Low SI ^4^ (≤9.83) *n* = 25	High SI ^4^ (>9.83) *n* = 19	*p* ^5^	Low SI ^4^ (≤9.83) *n* = 14	High SI ^4^ (>9.83) *n* = 25	*p* ^5^	
dp-ucMGP	+10.0 ± 23.7	+5.5 ± 18.7	0.75	−2.1 ± 23.0	+7.6 ± 22.8	0.62	0.17
Intima–media thickness	+2.6 ± 11.7	+0.5 ± 8.9	0.95	−0.5 ± 9.8	−0.5 ± 10.0	1.00	0.65
Diameter of the CA ^2^	−0.6 ± 3.1	+1.5 ± 5.5	0.20	−0.8 ± 2.6	+2.2 ± 5.3	0.12	0.62
Blood pressure at brachialis syst	−4.8 ± 19.9	+2.4 ± 12.5	0.28	+5.7 ± 10.6	+0.7 ± 7.1	0.43	0.056
**Blood pressure at brachialis diast**	**−2.8 ± 4.7**	**+6.0 ± 9.4**	**0.005**	+3.0 ± 8.3	+2.4 ± 9.6	0.84	**0.014**
Local pulse wave velocity CA ^2^	+5.8 ± 18.0	+1.4 ± 15.7	0.48	+11.7 ± 18.2	+6.8 ± 11.8	0.48	0.96
Distensibility coefficient	−3.8 ± 30.1	+4.3 ± 34.2	0.52	−13.2 ± 29.4	−10.4 ± 18.2	0.79	0.69
Compliance coefficient	−5.5 ± 27.2	+6.1 ± 33.8	0.37	−14.8 ± 31.5	−10.2 ± 20.3	0.65	0.60
**Young’s modulus**	**−16.8 ± 36.9**	**+42.5 ± 63.1**	**0.010**	+39.5 ± 76.0	+54.7 ± 79.5	0.66	0.15
Stiffness index	+16.3 ± 37.8	−6.1 ± 20.5	0.076	+15.4 ± 30.0	+3.6 ± 20.4	0.34	0.43
Blood pressure at CA ^2^ syst	−0.3 ± 8.7	+4.7 ± 14.2	0.35	+5.7 ± 10.8	+2.4 ± 7.6	0.55	0.079
**Blood pressure at CA ^2^ diast**	**−2.8 ± 4.6**	**+6.0 ± 9.4**	**0.004**	+3.0 ± 8.4	+2.3 ± 9.5	0.79	**0.011**
PWV ^3^ between CA ^2^ and radialis	+0.2 ± 15.5	−0.3 ± 12.1	0.90	+1.9 ± 11.7	+4.7 ± 10.8	0.84	0.59
PWV ^3^ between CA ^2^ and femoralis	+2.5 ± 15.0	+5.9 ± 16.3	0.72	+2.6 ± 13.3	+16.2 ± 21.1	0.081	0.22
Bone mineral density total body	+0.2 ± 1.8	+0.2 ± 1.6	0.99	−4.9 ± 15.8	−0.1 ± 1.6	0.070	0.12

^1^ Data are presented as relative change from baseline (in %). ^2^ Carotid artery. ^3^ Pulse wave velocity. ^4^ Stiffness index at baseline. ^5^ *p*-values for adjusted for multiplicity testing with Benjamini–Hochberg method. ^6^ Interaction *p*-value from ANOVA model with menopausal status and stiffness index status at baseline as fixed effects.

**Table 4:** Cardiovascular parameters’ evolution for women supplemented with MK-7.

Cardiovascular Parameter ^1^	Pre/Peri Menopausal Women			Post-Menopausal Women			Int *p* ^6^
	Low SI ^4^ (≤9.83) *n* = 20	High SI ^4^ (>9.83) *n* = 14	*p* ^5^	Low SI ^4^ (≤9.83) *n* = 21	High SI ^4^ (>9.83) *n* = 26	*p* ^5^	
dp-ucMGP	−32.7 ± 14.9	−30.1 ± 19.0	0.90	−31.0 ± 23.4	−23.2 ± 20.3	0.58	0.58
**Intima–media thickness**	+1.5 ± 13.4	−7.1 ± 11.6	0.16	−3.3 ± 11.4	−0.8 ± 6.9	0.48	**0.029**
Diameter of the CA ^2^	−0.1 ± 3.6	−0.01 ± 3.2	0.96	+0.7 ± 3.8	−1.3 ± 2.8	0.32	0.19
**Blood pressure at brachialis syst**	+1.6 ± 8.2	+1.9 ± 9.4	0.94	**+6.3 ± 10.4**	**−3.0 ± 9.0**	**0.007**	**0.027**
Blood pressure at brachialis diast	+2.5 ± 8.6	+4.3 ± 11.2	0.75	+4.2 ± 8.3	+1.2 ± 8.9	0.75	0.26
Local pulse wave velocity CA ^2^	+7.8 ± 12.5	−0.2 ± 9.7	0.14	**+8.2 ± 16.8**	**−3.3 ± 13.7**	**0.028**	0.60
Distensibility coefficient	−10.6 ± 20.1	+3.2 ± 22.4	0.23	**−9.7 ± 24.0**	**+13.3 ± 32.3**	**0.013**	0.44
Compliance coefficient	−12.9 ± 20.1	+0.6 ± 21.3	0.25	**−10.3 ± 23.6**	**+12.7 ± 32.6**	**0.012**	0.43
Young’s modulus	−11.6 ± 46.8	+45.5 ± 80.0	0.090	+0.4 ± 73.7	+17.7 ± 62.8	0.48	0.20
Stiffness index	+7.8 ± 23.9	−8.0 ± 15.7	0.069	+9.7 ± 26.0	−6.3 ± 20.8	0.069	0.99
**Blood pressure at CA ^2^ syst**	+3.3 ± 10.0	+6.4 ± 17.6	0.60	**+5.8 ± 9.7**	**−4.5 ± 11.7**	**0.026**	**0.017**
Blood pressure at CA ^2^ diast	+2.4 ± 8.6	+4.2 ± 11.3	0.77	+4.3 ± 8.3	+1.2 ± 8.9	0.77	0.25
PWV ^3^ between CA ^2^ and radialis	−3.3 ± 10.1	+0.7 ± 10.8	0.88	−0.6 ± 12.6	+1.2 ± 10.2	0.88	0.68
PWV ^3^ between CA ^2^ and femoralis	+4.5 ± 31.8	+5.4 ± 13.7	0.91	+0.1 ± 18	+3.4 ± 15.8	0.91	0.81
Bone mineral density total body	−0.3 ± 1.4	+0.4 ± 2.0	0.94	−0.2 ± 1.6	−1.8 ± 10.8	0.88	0.43

^1^ Data are presented as relative change from baseline (in %). ^2^ Carotid artery. ^3^ Pulse wave velocity. ^4^ Stiffness index at baseline. ^5^ *p*-values for adjusted for multiplicity testing with Benjamini–Hochberg method. ^6^ Interaction *p*-value from ANOVA model with menopausal status and stiffness index status at baseline as fixed effects.

## Data Availability

The raw data supporting the conclusions of this article will be made available by the authors on request.

## Abbreviations

The following abbreviations are used in this manuscript:

**Abbreviation**	**Definition**	**Equation**
IMT	Intima Media Thickness	
SBP	Systolic blood pressure	
DBP	Diastolic blood pressure	
d	Diameter of the carotid artery end-diastole	
Δd	The change in diameter from diastole to systole	
*p*	Pulse pressure in branchial artery	
Δp	The difference between systolic and diastolic blood pressure	SBP-DBP
DC	Distensibility coefficient	DC = (2 × d × Δd + Δd2)/(d2 × Δp)
CC	Compliance coefficient	CC = π (2 × d × Δd + Δd2)/(4 × Δp)
E	Young’s elasticity modulus	E = d/(IMT × DC)
β	Stiffness Index β	β = d × ln (SBP/DBP)/Δd
cPWV	Local carotid pulse wave velocity	1/**√** (ρ × DC) (ρ = blood density = 1060 kg/m^3^)
